# Psychosocial and contextual correlates of opioid overdose risk among drug users in St. Petersburg, Russia

**DOI:** 10.1186/1477-7517-6-17

**Published:** 2009-07-24

**Authors:** Lauretta E Grau, Traci C Green, Mikhail Torban, Ksenia Blinnikova, Evgeny Krupitsky, Ruslan Ilyuk, Andrei P Kozlov, Robert Heimer

**Affiliations:** 1 Department of Epidemiology and Public Health, Yale School of Public Health, 60 College St., New Haven, CT 06520-8034, USA; 2 Department of Addictions, Bekhterev State Research Psychoneurological Institute, 3, Bekhtereva Street, St. Petersburg 192019, Russia; 3 University of Alabama at Birmingham, School of Public Health, RPHB 330 1530, 3rd Avenue South, Birmingham, AL 35294, USA; 4 The Biomedical Center, 8, Vyborgskaya Street, St. Petersburg, Russia

## Abstract

**Background:**

Opioid overdose in Russia is a problem that has grown more severe as heroin abuse expanded over the past decade, yet few studies have explored it in detail. In order to gain a clearer understanding of the situation, 60 drug users, both in and out of drug treatment in St. Petersburg, were interviewed concerning their overdose experience and knowledge about overdose recognition and prevention.

**Methods:**

Using a semi-structured interview, we sought to identify and describe local attitudes, knowledge and experience (both self-sustained and witnessed) of opioid overdose. Bi-variate and multiple logistic regressions were performed in order to identify the relationship between overdose experience and sociodemographic factors, risk behaviors, and clinical psychiatric measures.

**Results:**

We found that having experienced or witnessed an opioid overdose within the previous year was common, overdose knowledge was generally high, but nearly half the participants reported low self-efficacy for effectively intervening in an overdose situation. In bivariate analyses, self-reported family problems (i.e., perceived problematic family interactions) were positively associated with both experiencing (t_56 _= 2.49; p < 0.05) and with witnessing a greater number of overdoses in the previous year (rho_s _= 0.31; p < 0.05). Having previously overdosed [Adjusted Risk Ratio (ARR) 1.7, 95% Confidence Interval (CI) 1.1–2.6] and higher SCL-90-R somatization scores (ARR 1.2, 95% CI 0.96 – 1.5) were independently associated in multivariable analyses with self-sustained overdose experience in the past year. Greater perceived likelihood of experiencing a future overdose and concern about medical problems were independently associated with witnessing a higher number of overdoses within the previous year. Over two thirds of the participants expressed interest in receiving training in overdose prevention and response.

**Conclusion:**

Opioid overdose experience is very common among drug users in St. Petersburg, Russia, and interest in receiving training for overdose recognition and prevention was high. Future research should target the development of effective overdose recognition and prevention interventions, especially ones that include naloxone distribution and involve drug users' families.

## Background

Since the 1990s, illegal drug use in the Russian Federation has increased dramatically [1]. By 2005 there were over 500,000 registered drug users in Russia, with estimates for the total number of drug users as high as six million for that year . Opium production in nearby Afghanistan provides many Russian cities with an accessible and bountiful source of heroin, which is administered primarily by injection [2-4]. Consequently, injection-associated infections such as HIV and hepatitis C virus are increasing among Russian drug users [5-7]. While the increases in injection-associated infections are striking, the annual estimated mortality rate attributed to drug overdose and problems related to drug use exceeds 100,000 [8] and surpasses mortality rates for HIV/AIDS [5,7].

There are few published Russian epidemiologic studies of opioid overdose. They tend to be regional studies and may therefore lack specificity in local opioid overdose prevalence rates and associated risk factors [9,10]. One large, multi-city study of drug users reported that more than 80% had ever witnessed an overdose of which 15% had been fatal [10]. Overdose fatality rates among the general population in Russia are increasing according to the State Drug Control Committee which reported 70,000 drug-related deaths in 2004 and 100,000 in 2005. Data from a St. Petersburg study of 520 injectors reported an annual overdose mortality rate of 2.1 per 100 person years [11] – almost double the reported rates for some Western European countries [12,13] – yet overdose prevention campaigns are rare. Building on the recognition of the large impact of opioid overdoses in St. Petersburg, we sought to identify and describe the contextual and psychosocial factors related to the outcome of experiencing and witnessing opioid overdose among a sample of 60 drug users.

## Methods

### Recruitment of Study Population

Inclusion criteria for participation in the study included anyone who was at least 18 years of age and had a history of illicit opioid use (past 30 days or prior to entry into drug treatment). Participants were recruited in St. Petersburg, Russia from June to October 2006 at one of two study sites. Thirty patients who had entered substance abuse treatment within the previous six weeks were recruited at the State Narcological Hospital (SNH). An additional 30 out-of-treatment opioid users were recruited at the Biomedical Center (BMC), a private non-profit biomedical research institution. Participants were recruited as a convenience sample. The targeted sample size of 60 was based upon the ability to detect a past-year opioid overdose prevalence of ≥45% with 80% power and 5% error; it was also considered to be a realistic and feasible sample size to recruit within the timeframe of this pilot study. Efforts were made to recruit at times of highest potential participant availability (i.e., during the daytime at SNH and evenings at BMC). The study was approved by the Yale University Human Investigations Committee and the institutional review boards at the two St. Petersburg sites. All participants provided informed consent prior to data collection and were remunerated with gifts equivalent to US$10 for participating.

### Study Procedures

Each participant completed a face-to-face interview with a trained interviewer. Time to complete the survey was approximately 60–90 minutes. The instrument, specifically developed for this study, included a series of questions covering (1) sociodemographic factors, (2) knowledge about overdose symptoms, risk factors, and prevention strategies, (3) self-reported history of having witnessed overdoses and details about the most recently witnessed overdose, and (4) self-reported history of having personally experienced an overdose and details of their most recent overdose.

Four open-ended questions assessing recall knowledge of overdose symptoms, risk factors, and prevention strategies appeared at the beginning of the interview and before asking about overdose experience. These four items typically elicited short responses (e.g., responses for opioid overdose symptoms included "cyanosis", "blue lips/face", "not breathing", "unresponsive"). Each response was coded for accuracy (accurate/inaccurate) based upon the current scientific understanding of overdose symptoms, risk factors, and preventive measures (e.g., not injecting alone, avoid using alcohol or other central nervous system depressants in combination with opioids, injecting a small preliminary dose to judge the strength of the drug). Responses were independently coded by two researchers (TCG, KB) for content and accuracy level, and the final content and accuracy codes were established by consensus (LEG, TCG, MT, KB).

Prior to asking a series of forced-choice questions about the history and details of witnessed or self-sustained overdoses, the following description of overdose symptoms was provided in order to promote consistency in reporting: "There are two different types of overdoses. The symptoms are as follows: 1. Amphetamine overdose: the person is 'going crazy' (psychosis), 'shakes' or seizures, racing heartbeat, severe sweating or clammy body. 2. Opioid/heroin overdose: pale or blue skin, shallow or infrequent breathing, loss of consciousness, insensitivity to pain, no response to shaking or calling the person's name."

The final section of the instrument included items from the Addiction Severity Index (ASI) [14-16] and the Symptom Checklist-90 (SCL-90-R) [17-20]. The ASI and SCL-90-R are multidimensional, self-report measures that are frequently used in clinical and research settings to evaluate and monitor potential problems salient to mental health and substance abuse treatment. The ASI assesses the perceived severity of problems and need to seek professional help for each of seven domains (i.e., medical, employment, alcohol, drug use, legal, family, and psychiatric) such that higher scores signify greater perceived problems for the given domain. Only those ASI items that were necessary for generating the seven composite scores were included in our survey. The SCL-90-R assesses psychological symptoms within nine domains (i.e., somatization, obsessive-compulsive, interpersonal sensitivity, depression, anxiety, hostility, phobic anxiety, paranoid ideation, and psychoticism) as well as quantifying a person's overall level of psychological distress (i.e., global severity index). The Russian version of the SCL-90-R has not been normed, and clinical cutoff scores do not exist. However, higher scores are indicative of greater perceived distress concerning the given domain.

### Data Analyses

Data were entered into a Microsoft Access database and exported to SPSS version 12.0 for analysis. The primary outcomes were recent (past 12 months) self-sustained overdose experience and number of recently witnessed overdoses. Lacking normative scores for the Russian SCL-90-R, we compared subscale scores within and across individuals. ASI composite scores were calculated for five of the seven domains. The medical and alcohol composites could not be calculated due to a data collection error in which two items that are necessary for computing composite scores were inadvertently omitted from the Russian instrument; for these two subscales, item level analyses were conducted instead.

Descriptive statistics were calculated to characterize the study sample. Bivariate analyses to identify possible associations of demographic, psychosocial, and contextual factors with the two outcome variables were performed. All associations with alpha ≤ 0.1 are reported.

Exploratory regression analyses were conducted to examine the association between independent variables and the outcomes of recent self-sustained overdose and the number of recently witnessed overdoses. Due to the high prevalence of recent self-sustained overdose, we used the relative risk regression approach to avoid overestimation of associations that may occur when the rare disease assumption needed for logistic regression is not satisfied [21]. Since witnesses of more and recent overdoses may have the most impact on reducing overdose rates, we modeled counts of recently witnessed overdoses (past 12 months) to help elucidate characteristics of a target population for future overdose prevention training interventions. Thus, a negative binomial regression of the number of recently witnessed overdoses was estimated. Correlates of the overdose outcomes were those with significant association (p ≤ 0.10) in the bivariate analyses, and we also controlled for potential confounders (e.g., site). The model building proceeded using backward and forward approaches in a non-automated fashion. Parsimony guided the final model decision in the interest of conserving statistical power.

## Results

### Sample Characteristics

The study sample (*n *= 60) consisted primarily of young, male, injection drug users (Table 1). The majority of participants resided with partners or parents, with only 21.7% living alone. More than half of the sample had university or specialized professional schooling (55.0%), but only 26.7% were currently employed. Geographic distribution of residence was fairly even across the four quadrants of the city, and few (< 7%) resided beyond the city limits.

**Table 1:** Study population socio-demographics and overdose experience

**Variable**	**N (%)**	**Mean (SD)**
Site		
State Narcological Hospital	30 (50)	
Biomedical Center	30 (50)	

Age in years [mean (SD)]	30.8 (7.5)	

Male	37 (61.7)	

Age at first opioid use [mean (SD)]	20.3 (5.8)	

Years from first opioid use to first opioid overdose [mean (SD)]	4.5 (6.0)	

Living situation		
Alone	13 (21.7)	
Parents	26 (43.3)	
Spouse	13 (21.7)	
Sex partner	2 (3.3)	
Relatives	5 (8.3)	
With friends	1 (1.7)	

Highest level of education		
Did not complete middle school	11 (18.3)	
Completed middle school	16 (26.7)	
Special middle (PTU/tech school)	18 (30.0)	
Institute//university	15 (25.0)	

Currently employed	16 (26.7)	

District of inhabitance		
Central	12 (20.3)	
South	15 (25.4)	
North	17 (28.8)	
East	11 (18.6)	
Suburbs	6 (6.8)	

Addiction Severity Index		
Legal composite		0.13 (0.17)
Family composite		0.38 (0.21)
Psychiatric composite		0.39 (0.24)
Drug composite		0.14 (0.10)
Employment composite		0.75 (0.26)
Medical, items:		
Days bothered by medical problems*		10.53 (10.35)
How troubled by medical problems**		2.13 (1.29)
Alcohol, items:		
Days used alcohol*		4.61 (7.71)
Days used alcohol to intoxication*		1.71 (3.48)
Days experiencing problems with alcohol*		3.10 (7.22)
How troubled by alcohol problems**		0.77 (1.17)
Importance of getting treatment for alcohol problems**		0.97 (1.46)

SCL-90 Mean score (SD)		
Global Severity Index		1.24 (0.69)
Obsessive-compulsive		1.79 (1.09)
Interpersonal sensitivity		1.45 (0.87)
Depression		1.25 (0.94)
Anxiety		1.35 (0.95)
Hostility		1.39 (0.94)
Phobic anxiety		0.82 (0.85)
Paranoid ideation		1.13 (0.88)
Psychoticism		0.70 (0.76)
Somatization		1.31 (0.82)

Ever injected	59 (98.3)	
Past 30 days, injected	29 (49.2)	
Heroin	29 (100)	
Opioids	4 (13.8)	
Amphetamines	2 (6.9)	
Other	3 (10.3)	

Inject alone all or most of the time	17 (29.3)	

*in the past 30 days

**(0 = not at all 4 = extremely)

Relative to the other ASI subscales, the employment, psychiatric, and family composite scores were the highest. ASI item-level findings revealed that medical problems were common, with an average of 10 days of medical problems reported in the previous 30. By contrast, alcohol use and problems related to alcohol were reported infrequently. The SCL-90 scores were lowest for the psychoticism subscale and highest for the obsessive-compulsive and interpersonal sensitivity subscales.

Half the sample was recruited from a hospital-based treatment site (SNH), and consequently only five of these participants (16.7%) had injected drugs in the past month (all Russian drug treatment is abstinence-based); all had injected within the past six weeks, however. All participants not in hospital-based treatment (BMC) reported injecting heroin in the past 30 days. Almost three quarters of the sample reported injecting heroin at their most recent injection (74.6%) or injecting with others all or most of the time (70.7%).

There were several differences in the sample according to recruitment site. The SNH sample was younger (26.7 years vs. 35.0 years, p < 0.001), initiated opioid use at a younger age (17.7 years vs. 22.7 years, p < 0.001), and experienced their first opioid overdose at a younger age (21.2 years vs. 27.5 years, p < 0.005) than the BMC sample. SNH participants were less likely to have witnessed an overdose within the previous year (53.3% vs. 79.3%, p < 0.05) but were more likely to report having been present when the overdose victim died or woke up (82.1% vs. 10.3%, p < 0.0001). Study site differences were controlled for in the subsequent regression analyses.

### Overdose Knowledge and Attitudes

Participants accurately described opioid overdose symptoms, with 86.7% of the sample mentioning one or more actual symptoms; most frequently mentioned were cyanosis (56.7%), loss of consciousness (53.5%), and absence of breathing (51.7%). In contrast, only 21.7% of respondents provided correct amphetamine overdose symptoms; 46.7% of respondents reported not knowing any symptoms. Participants correctly identified key risk factors for opioid overdose such as taking too large a dose (24.4%), mixing drugs with alcohol (21.1%), and variability of drug quality (17.8%). However, misinformation about overdose risk factors was observed; "bad self-control" or a flawed character was mentioned by approximately one in five individuals. Effective overdose prevention strategies identified by participants included a preliminary injection or "tasting" of a small quantity of drug (38.3%) and not mixing drugs and alcohol (13.3%). Less effective or ineffective strategies were also reported: knowing one's optimal dose (31.7%) and judging the physical appearance of the drug (3.3%). Eight participants (13.3%) failed to mention any strategy. Abstinence (6.7%) and better self-control (3.3%) were also noted as effective, albeit nonspecific, strategies for reducing overdose risk.

Nearly half of participants (41.4%) reported lacking confidence in or being unsure of their ability to help in an overdose situation. They expressed interest in receiving information about overdose prevention (76.3%) or training on how to respond to an opioid overdose (67.2%). Less than half of the sample (44.1%) had heard of naloxone despite its availability by prescription at pharmacies in Russia and its use in hospitals and some emergency response service units.

### Reports of Overdoses in the Community

Reports of overdoses, both fatal and nonfatal, were common. Participants reported having heard of a median of five (range = 0 – 60) non-fatal overdoses and of two (range = 0 – 30) fatal overdoses in the past year alone. Almost two thirds of participants (63.3%) reported that all or most of their friends had ever overdosed. Fifteen respondents (25.0%) rated their risk of personally overdosing as somewhat or extremely likely during the next year, and a similar proportion were somewhat or extremely concerned about this risk (28.3%). Participants were more concerned about their peers' potential risk for overdose than their own (50% vs. 28.3%).

### Self-sustained Overdose Experience

**Figure 1:**
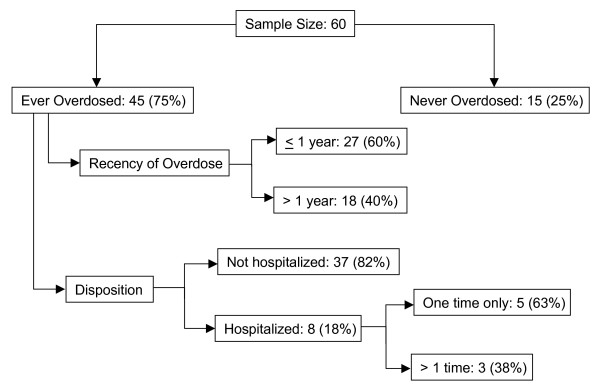
**Prevalence, recency and disposition of self-sustained overdoses in a sample of 60 opioid abusers in St. Petersburg, Russia**.

Three quarters of participants had personally experienced an opioid overdose in their lifetime, and the median lifetime number of overdoses was 4 (25, 75 percentile: 2, 10). The median age at first overdose was 21.5 years (25^th^, 75^th ^percentile: 19, 27), and the mean time from first opioid use to first overdose was 4.5 years (SD 6.0). Most participants who reported having ever overdosed had experienced at least one within the previous year (60.0%, or 45.0% of total sample). Eight participants (17.8%) had ever been hospitalized following their overdose, usually only once (62.5%) (Figure 1).

Heroin was the most commonly reported drug used at last overdose (95.6%), with alcohol (11.1%) or other opioids (8.9%) reported less frequently. Immediately prior to their last overdose, some participants had been in drug treatment (24.4%) or incarcerated (18.2%). Most overdoses occurred at home (42.2%), at a friend's home (17.8%), or on the street (20.0%). Others were usually present (77.8%) at participants' last overdose and attempted resuscitation procedures over half (60.0%) the time. No resuscitation was attempted in 8.9% of cases in which others were present. The most commonly reported resuscitation procedure was physical stimulation (36.5%) such as slapping, walking around, applying cold water or ice. Medical intervention (e.g., CPR/rescue breathing or administering naloxone) was reported in 33.3% of cases. Less effective resuscitation activities (e.g., saline or milk injection) were performed in 6.3% of cases. Of those who recalled what happened to them at their last overdose, five people reported receiving medical attention (11.1%), and two were taken to the hospital for a one-day stay. Police did not arrive at any of the last reported self-sustained overdoses.

### Factors Associated with Recent Self-Sustained Overdose

Recent overdose was defined as one that occurred in the past year. Correlates of recent overdose (Table 2) included a greater number of lifetime previous overdoses (median of 5), a higher SCL-90 subscale score for somatization, stronger expectations of personally experiencing another overdose, and higher ASI family composite scores, reflecting greater perceived problematic family interactions. Examination of family subscale items revealed that people reporting problematic interactions with their mothers – as opposed to other family members – were at risk of experiencing a recent overdose. They were also more troubled by their family problems and felt it more important to seek help for these family problems than did participants who had not experienced a recent overdose. An exploratory relative risk regression revealed two independent correlates of having a recent self-sustained overdose: having previously overdosed and higher scores on the somatization subscale (Table 2).

**Table 2:** Variables Associated With recent experience of an opioid overdoses

	**Bivariate Analyses**	**Relative Risk Regression**	
		
		Adjusted Risk Ratio	95% Confidence Interval
Previously overdosed	χ^2 ^= 14.6 ***	1.7	1.1 – 2.6

Number of overdoses experienced	U = 142.50***Z = -4.33		

Perceived Likelihood of overdosing again	χ^2 ^= 5.72*		

Higher SCL-90 Somatization score	t_58 _= 2.50**	1.2	0.96 – 1.5

Higher ASI Family composite score	t_56 _= 2.49*		

Problems with mother	χ^2 ^= 4.42*		

Troubled by family problems	t_57 _= 2.26*		

Importance of getting help for family problems	t_57 _= 2.50*		

* p < 0.05

** p < 0.01

*** p < 0.001

### Witnessing overdoses

**Figure 2:**
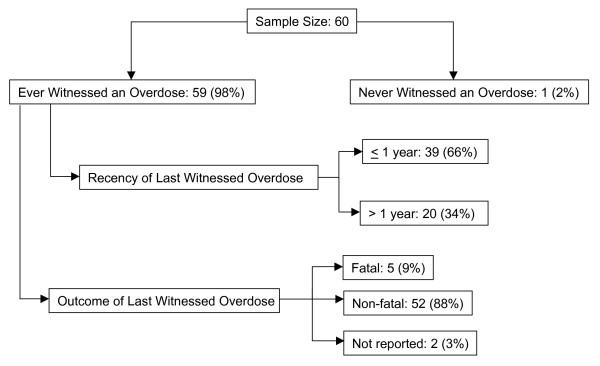
**Prevalence, recency and reported outcomes of witnessed overdoses in a sample of 60 opioid abusers in St. Petersburg, Russia**.

All but one participant had ever witnessed an overdose (98.3%), and two thirds (66.1%) had witnessed at least one within the previous year (median 2; 25, 75 percentile: 1, 4; Figure 2). Altogether, participants reported witnessing a total of 226 overdoses in the past year. In only 20.8% of these instances was an ambulance called. The most commonly reported reasons for not calling an ambulance were: confidence in ability to resuscitate without medical intervention (54%), fear of police (14.3%), and lack of confidence in the ambulance response (11.4%).

When asked about the last witnessed overdose, regardless of its recency, most participants had been present when the overdose victim had taken drugs (82.8%), when the person became distressed (86.2%), and when the person became unconscious (96.6%). By contrast, fewer participants reported being present at the time of medical response or ambulance arrival (49.2%) or at the end when the person died or was revived (45.6%), suggesting that leaving an overdose scene is common. The overdose victim died in five (8.6%) of the last witnessed overdoses (Figure 2). All victims had used heroin, primarily by injection (91.5%); 59.3% had also used alcohol; 8.5% had also used amphetamines; and three cases had reportedly ingested some other substance in addition to heroin. The overdoses tended to occur in private settings, primarily at home (30.5%) or at a friend's home (25.4%), but many occurred in public places (25.4%). The most commonly perceived causes of the last witnessed overdose were combined drug and alcohol use (32.2%), purity of the drug (28.8%), a recent period of abstinence (11.9%), or recent release from either drug treatment or incarceration (8.5%).

It was the norm that multiple persons witnessed overdoses. On average, 3.2 people (SD 3.1, median = 2) were present at the last witnessed overdose. In most instances (86.4%), witnesses were drug users. Fellow drug users were also listed most often (79.7%) as a resuscitator at the last witnessed overdose. Hospital (10.2%) and ambulance (6.8%) staff were also mentioned as resuscitators. Multiple resuscitation strategies were typically employed. The most commonly reported strategies attempted during the last witnessed overdose were physical stimulation (67.8%) and CPR/rescue breathing (57.7%). Participants reported that an ambulance was called in 37.3% of the last witnessed overdoses and arrived in a median of 20 minutes (range 5 – 60). Naloxone was administered in five cases (22.7%), two of which did not involve a request for an ambulance.

Variables associated with having witnessed more overdoses in the past year were being more concerned about personal and others' risk of overdose, higher expectation of future self-sustained overdose, more reported days with medical problems in the past month, being more troubled by medical problems, and higher scores on the SCL-90-R somatization subscale and ASI family and drug composite scores. In the negative binomial regression (controlling for site), having higher expectations of future self-sustained overdose and being more troubled by medical problems in the past month were associated with having witnessed a greater number of recent overdoses (Table 3).

**Table 3:** Variables associated with recent witnessing of an opioid overdoses

	**Number of Witnessed Overdoses (past year)**	**Negative Binomial Regression**	
		
		Adjusted Parameter Estimate	95% Confidence Interval
Greater perceived likelihood of overdosing again	ρ_s _= .43***	0.3	0.02 – .50

Greater concern for self-sustained overdose risk	ρ_s _= .33**		

Greater concern for others' overdose risk	ρ_s _= .32**		

Higher SCL-90 Somatization score	ρ_s _= .40***		

Higher ASI Drug composite score	ρ_s _= .37**		

Higher ASI Family composite score	ρ_s _= .31*	0.7	-0.9 – 2.4

More days with medical problems^a^	ρ_s _= .32**		

More troubled by medical problems^a^	ρ_s _= .42***	0.6	0.3 – 0.8

* p < 0.05

** p < 0.01

*** p < 0.001

^a ^Past 30 days

ρ_s _= Spearman correlation coefficient

## Discussion

Our study is the only one to date that examines opioid overdose risk among St. Petersburg drug users and the first to explore the relationship between opioid overdose experience and psychological screening measures that are used in Russian clinical settings. Most overdoses involved heroin and occurred in private residential settings. Similar to other regional studies conducted in Central and Eastern Europe and the former Soviet Union [9,10], we found that witnessing or personally experiencing an opioid overdose was very common. In contrast to other overdose studies in Australia, Great Britain, and the United States [22-26], a higher proportion of our participants had experienced at least one overdose within the previous year, and the lifetime number of overdoses was substantially greater. Larger scale studies of drug users in St. Petersburg are needed to confirm if these observations are generalizable.

Our findings also indicated that participants were quite knowledgeable about opioid overdose symptoms. However, misinformation and gaps in knowledge exist. For example, "knowing one's optimal dose" was cited by almost one quarter of the sample as an effective overdose prevention strategy. Overdose prevention programs should inform drug users that this strategy is impossible in practice when using heroin since drug strength can vary substantially. The study findings also indicated that St. Petersburg drug users tended to overdose within four years of initiating opioid use. This suggests the importance of reaching individuals early in their drug use careers and educating them about overdose risk, recognition, and prevention. Participants were less knowledgeable about stimulant overdose than about opioid overdose. This observation, coupled with the reported increases in cocaine and stimulant use in Russia [27], suggests the need for studies on the prevalence of stimulant overdose, its prevention, and the development of effective community-based response interventions. Furthermore, requests for an ambulance were infrequent. It is hypothesized that this observation may be a function of drug users' skepticism about the effectiveness of the emergency response system and concerns about potential police involvement [28].

The strongest correlates of experiencing a recent overdose were previous self-sustained overdose experience followed by a higher SCL-90-R somatization subscale score. One hypothesis to account for the first finding is that people tended to continue to engage in behaviors that increased their risk of overdose (e.g., combined use of opioids and alcohol). The finding concerning the SCL-90-R somatization subscale suggests that suboptimal health status may place individuals at risk of overdose. The somatization subscale assesses the perceived level of physical distress (e.g., headache, pains, numbness) such that a person suffering from physical problems will score higher than someone with little or no physical complaints. Since we did not collect health status data, it is possible that the subscale may have served as an indirect measure of underlying illness. Infections such as HIV or hepatitis C, both of which are endemic among Russian injectors [29,30], interfere with optimal immune functioning and metabolism and place individuals at increased risk of drug overdose [31].

Greater perceived likelihood of overdosing in the future and concerns about personal medical problems were the strongest correlates of witnessing more overdoses recently. One hypothesis to account for the association between perceived likelihood of future overdose and witnessing multiple overdoses recently is that the act of witnessing may heighten one's awareness of the pervasiveness of overdose and sense of fatalism about the future. In addition, drug users who have recently witnessed multiple overdoses may be ideal candidates for an overdose prevention intervention and may have a strong impact on reducing overdose rates within their community. Additional research is needed to clarify these issues.

Several limitations in this study should be noted. First, the potential generalizability of the findings is limited by the study's non-random sampling strategy. Although we sampled at two different venues, drug users not seeking treatment or unwilling to participate in research studies may be under-represented. Second, given the relatively small sample size and limited power, regression analyses should be interpreted with caution and viewed as exploratory in nature. We attempted to limit the number of variables included in the regressions to those permissible for our sample size, but relationships should optimally be tested with a larger, more representative sample. The cross-sectional nature of this study does not permit determination of causal associations. Finally, only self-reported data were collected and therefore are open to the vulnerabilities of social desirability bias, interviewer bias, and recall bias. We made every effort to be empathic and non-judgmental and to provide specific definitions for opioid overdose and stimulant overdose in order to reduce measurement error in reporting. Nevertheless, it is possible that biases may have influenced our findings.

## Conclusion

The observation that virtually all respondents had opioid overdose experience (both direct and indirect) speaks to the magnitude of the problem in St. Petersburg. Expectations about respondents' and their drug-using friends' strong likelihood of overdosing in the future provide evidence of their awareness of and concern about overdose risk. Drug users noted their lack of confidence in being able to respond to overdose situations and were interested in receiving training on overdose prevention, recognition, and response. This study highlights the need for and potential receptiveness to an overdose prevention program. The results also suggest that the involvement of family members in drug treatment and overdose prevention programs may also be effective in reducing opioid-related harms.

## Competing interests

The authors declare that they have no competing interests.

## Authors' contributions

LEG participated in developing the study design, helping to create the study instruments, performing data analyses, and writing the manuscript. TCG participated in developing the study design, helping to create the study instruments, performing data analyses, and writing the manuscript. MT participated in developing the study design, helping to create the study instruments, performing data analyses, conducting the interviews, and writing the manuscript. KB participated in developing the study design, helping to create the study instruments, performing data analyses, conducting the interviews, and writing the manuscript. EK supervised the conduct of the study at the Bekhterev Institute and provided final approval of the manuscript for the study site. RI supervised all interviews conducted at the Bekhterev Institute. APK supervised the conduct of the study at the Biomedical Center Institute and provided final approval of the manuscript for the study site. RH conceived of the study and contributed to the writing of the manuscript. All authors read and approved the final manuscript.
